# Comparison of the Effects of Target-Controlled Versus Conventional Infusion Sedation on Recovery in Geriatric Patients Undergoing Diagnostic Cystoscopy

**DOI:** 10.4274/TJAR.2025.252107

**Published:** 2026-02-09

**Authors:** Nesibe Sena Bayburt, Fatma Nur Duruk Erkent, Ayşegül Güven, Neslihan Alkış

**Affiliations:** 1 Osmancık State Hospital, Clinic of Anaesthesiology and Reanimation, Çorum, Türkiye; 2 Ankara University Faculty of Medicine, Department of Anaesthesiology and Reanimation, Ankara, Türkiye

**Keywords:** Geriatric anaesthesia, propofol, sedation, target-controlled infusion, total intravenous anaesthesia

## Abstract

**Objective:**

Procedural sedation management in geriatric patients undergoing cystoscopy requires careful monitoring due to age-related physiological changes and increased sensitivity to anaesthetic agents. Although both target-controlled infusion (TCI) and conventional total intravenous anaesthesia (TIVA) techniques with propofol are commonly used methods for sedation, their comparative effectiveness and safety in this population remain subjects of ongoing investigation. This study aims to compare the effectiveness of the two techniques in terms of time to induction, recovery time, hemodynamic stability, airway intervention requirements, and propofol consumption.

**Methods:**

This prospective, randomized study enrolled 60 male patients aged 65 years and older who were scheduled to undergo elective cystoscopy. Participants were randomly assigned to either the TCI group (n = 30) or the TIVA group (n = 30). The two groups were compared in terms of induction time, recovery time, hemodynamic parameters, airway interventions, and total propofol consumption.

**Results:**

Compared with the TCI group, the TIVA group presented significantly shorter induction-to-surgery initiation and recovery times (*P*=0.009 and *P*=0.016, respectively). However, systolic blood pressure was more stable in the TCI group compared to the TIVA group (*P*=0.014). Propofol consumption per unit time was greater in the TIVA group (*P*=0.048), although total propofol usage did not differ significantly. Airway intervention was more common in the TIVA group, particularly in the early phase; however, this difference was not significant.

**Conclusion:**

Both TCI and TIVA are effective sedation techniques for geriatric cystoscopy. While TIVA provides faster induction and recovery, TCI offers better hemodynamic stability and may reduce propofol requirements. Further studies are recommended to confirm these findings in broader patient populations.

**Main Points**

• Sedation with total intravenous anaesthesia (TIVA) results in shorter induction and recovery times than does target-controlled infusion (TCI) in geriatric patients undergoing diagnostic cystoscopy.

• TCI provides more stable systolic blood pressure throughout the procedure.

• Although total propofol consumption was similar in both groups, propofol usage per unit of time was significantly greater in the TIVA group.

• Airway interventions were more frequently needed in the TIVA group, particularly during the early procedural period.

• The findings indicate that both TCI and TIVA are safe and effective anaesthetic approaches, as neither group experienced major postoperative complications.

## Introduction

Sedation management in the geriatric population is crucial for maintaining perioperative hemodynamic stability, ensuring a rapid recovery, and preserving cognitive function. Due to the increased sensitivity of geriatric patients to anaesthetic agents, they should be monitored more closely; furthermore, appropriate dose adjustments must be made accordingly.

Propofol is a widely used anaesthetic agent, commonly preferred for both induction and maintenance phases of anaesthesia. For maintenance, propofol may be administered via a conventional manually controlled total intravenous anaesthesia (TIVA) method or through target-controlled infusion (TCI) systems. In the conventional approach, clinicians manually adjust infusion rates using pharmacokinetic data obtained from prior clinical studies to reach the desired depth of anaesthesia. Conversely, TCI devices employ pharmacokinetic models that calculate and deliver specific infusion rates to achieve a predetermined drug concentration either in plasma or at the effect site, tailored to individual patient characteristics.^1^ These advanced infusion systems rely on three-compartment pharmacokinetic models, taking into account variables such as age, weight, sex, body height, tissue perfusion, and clearance rates. Once the target concentration is achieved, the TCI system maintains that level by adjusting the infusion rate automatically.^2^ TCI can be effectively applied in both sedation and general anaesthesia practices. Although several studies have investigated the use of TCI in gastrointestinal (GI) endoscopic procedures such as colonoscopy and upper GI endoscopy, limited data exist regarding its application in elderly patients undergoing cystoscopy.^3, 4^ Considering the growing elderly population and the frequency of such urologic diagnostic procedures, evaluating optimal sedation strategies is crucial. This prospective study aimed to compare the clinical performance of TCI and TIVA techniques in geriatric patients undergoing cystoscopy under sedation. Our primary focus was to assess and compare both methods in terms of induction time, recovery profile, hemodynamic stability, overall propofol usage, and airway support requirements.

## Methods

This study was conducted between December 1, 2022, and September 1, 2023, in the operating rooms of Ankara University Faculty of Medicine İbni Sina Hospital. The Human Research Ethics Committee of Ankara University Faculty of Medicine approved this study (approval no.: İ10-611-22, date: 10.11.2022). Sixty male patients aged >65 years, with American Society of Anesthesiologists (ASA) Physical Status I-III, scheduled for elective cystoscopy or urethrocystoscopy under sedoanalgesia, were included in the study. Patients were informed about participation in the study before the procedure, and written informed consent was obtained from all participants. Patients with ASA IV, those undergoing emergency surgery, female and pediatric patients scheduled for cystoscopy, patients aged <65 years, those requiring general anaesthesia due to procedural necessity, and those who did not provide informed consent were excluded. Additionally, patients whose procedure times were shorter than 7 minutes or longer than 12 minutes were excluded. The exclusion of cases with procedure durations shorter than 7 minutes or longer than 12 minutes was intended to minimize variability in propofol consumption and recovery time attributable to extreme procedural lengths. This approach was intended to obtain a more homogeneous sample and to enhance comparability between the groups.

Demographic characteristics such as age, body weight, height, sex, ASA classification, comorbid medical conditions, chronic medication use, and prior surgical history were systematically recorded for all participants. Standard ASA-recommended monitoring—comprising electrocardiography, pulse oximetry, and non-invasive blood pressure measurement (NIBP)—was initiated for all patients upon arrival in the operating room. Additionally, bispectral index (BIS) monitoring (Covidien, Ireland) was performed, and the baseline values were recorded. Intravenous access was established in all patients, and a crystalloid infusion was initiated. All patients received intravenous fentanyl at a dose of 0.5 micrograms per kilogram. Additionally, 1 mg kg^-1^ lidocaine was administered intravenously before the initiation of the propofol infusion. Following oxygen supplementation at 5 L min^-1^ via facemask, patients were randomly assigned to one of two groups: target-controlled infusion (TCI; n = 30) or TIVA (TIVA; n = 30).

In the TCI group, remifentanil was initiated at an infusion rate of 0.05 µg kg^-1^ min^-1^. Two minutes later, propofol infusion was initiated via a TCI device with the Schnider pharmacokinetic model, which targeted an effect-site concentration of 2 micrograms per milliliter. The time of infusion initiation was recorded for all patients. If necessary, the effect-site target concentration was increased in increments of 0.5 micrograms per milliliter until the Ramsay Sedation Scale reached level 6. Following the attainment of level 6 sedation, patients were placed in the lithotomy position and the surgical intervention was subsequently commenced. The time of surgical procedure initiation was recorded. After the procedure began oxygen saturation, heart rate, NIBP and BIS values were documented every two minutes. Episodes of desaturation and airway intervention requirements were also noted.

The TIVA group received an identical remifentanil infusion protocol (0.05 µg kg^-1^ min^-1^). Two minutes later, propofol was administered as a 0.5 mg kg^-1^ intravenous bolus, followed by continuous infusion at a rate of 50 µg kg^-1^ per minute via a perfusion pump. The initiation time of propofol infusion was noted. If required, additional intravenous boluses of 10 mg propofol were administered. Following the attainment of level 6 sedation, patients were placed in the lithotomy position, and the surgical intervention was subsequently commenced. The surgical procedure initiation time was documented. Following the commencement of surgery oxygen saturation, NIBP, heart rate and BIS values were measured and recorded every two minutes.

In both groups, sedation depth was titrated to achieve Ramsay Sedation Scale level 6. BIS monitoring was used as an adjunct to avoid oversedation, but BIS was not used as the primary target parameter. BIS values were recorded every 2 minutes during the procedure. Titration in propofol dosage was planned in the event that BIS values remained below 40 for more than 2 consecutive minutes. Throughout the procedure in both groups, if peripheral oxygen saturation levels dropped below 90%, the flow was increased, and airway maneuvers such as chin lifts were employed as needed. If desaturation persisted, an oropharyngeal airway was inserted. A mechanical ventilator was retained on standby for assisted ventilation with a facemask if needed. Atropine (0.5-1 mg) was prepared for administration in cases of bradycardia (heart rate <50 beats per minute), and a crystalloid infusion was initiated when the mean arterial pressure dropped below 60 mmHg. Ephedrine was prepared at a concentration of 5 mg mL^-1 ^and kept readily available on the table. Additionally, the presence of desaturation and the need for airway intervention were noted for all patients.

Infusions were stopped at the end of the surgical procedure in both groups. The total volume of propofol consumed during the procedure, the duration of surgery completion, and the final vital signs and BIS values at the end of surgery were recorded. The modified Aldrete recovery score was used to assess postoperative recovery. Patients who achieved a score of 9-10 were transferred from the operating room to the post-anaesthesia care unit. The time elapsed from the end of the surgical procedure to transfer to the recovery room was recorded. In the recovery room, patients were evaluated for postoperative nausea, vomiting, and delirium, and the findings were documented. All patients were closely monitored for the occurrence of major postoperative complications within the first 24 hours following surgery.

### Statistical Analysis

All statistical procedures were conducted using SPSS software version 11.5. Numerical data were described using both mean ± standard deviation and median with range (minimum-maximum), while categorical variables were expressed as frequencies and proportions. To compare a continuous variable between two independent categorical groups, the Student’s t-test was applied under the assumption of normality; if this assumption was not met, the non-parametric Mann-Whitney U test was used instead. Relationships between categorical variables were analyzed using either the chi-square test or Fisher’s exact test, depending on expected cell counts. For evaluating changes over time within and between groups for continuous outcomes, a two-way repeated measures ANOVA was utilized. Statistical significance was defined as a p-value less than 0.05. The sample size calculation determined that enrolling a total of 96 participants (48 per group for TIVA and TCI) would provide 80% statistical power to detect a medium effect size (Cohen’s d = 0.5). During the course of the study, several participants were excluded due to predefined exclusion criteria and unforeseen clinical circumstances, resulting in a smaller sample size than initially planned (48 participants per group). Therefore, the sample size was recalculated based on the final number of participants. In line with similar studies in the literature, the estimation was performed assuming a large effect size (Cohen’s d = 0.8) for the difference in emergence time between the TIVA and TCI groups, with a significance level of 0.05, a statistical power of 0.80, and using the Mann-Whitney U test.^5^ This recalculation demonstrated that a minimum of 27 participants per group (total n = 54) would be sufficient, confirming that the study retained adequate statistical power despite the reduced sample size.

## Results

**Figure 1:**
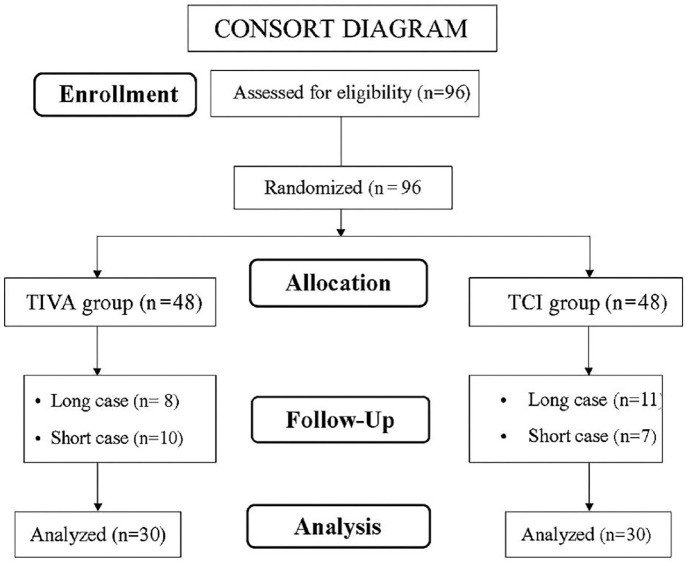
CONSORT flow diagram of patient distribution. CONSORT, Consolidated Standards of Reporting Trials; TCI, target-controlled infusion; TIVA, total intravenous anaesthesia.

A total of 96 patients were initially enrolled in the study. However, patients whose procedure duration was shorter than 7 minutes or longer than 12 minutes were excluded from the analysis. Statistical analyses were performed on a total of 60 patients, with 30 in the TIVA group and 30 in the TCI group (Figure 1).

When the demographic data were compared between the TIVA and TCI groups, a significant difference was found only in terms of hypertension diagnosis (*P=*0.018).  Hypertension was observed in 73.3% of patients in the conventional TIVA group and in 43.3% of those in the TCI group (Table 1).

**Figure 2:**
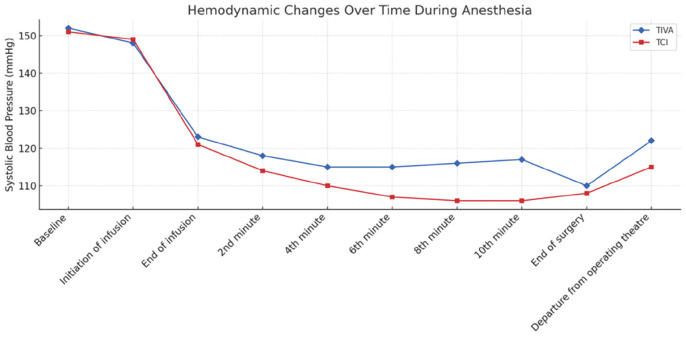
Graphical representation of the time-dependent changes in systolic blood pressure between the two groups. TCI, target-controlled infusion; TIVA, total intravenous anaesthesia.

A statistically significant difference was observed between the TIVA and TCI groups regarding the time-dependent trend of systolic blood pressure (SBP) (*P=*0.014). At each time point, the mean SBP value in the TIVA group was 0.964 units higher than that in the TCI group (Figure 2).

When the differences in blood pressure over time were evaluated between the TIVA and TCI groups, significant differences in SBP were observed at the 6^th^ and 8^th^ minutes and at the end of surgery and time of transfer from the operating table (*P=*0.027, *P=*0.011, *P=*0.011, and *P=*0.015, respectively). The measured SBP values at these time points were greater in the TIVA group than in the TCI group. The temporal patterns of oxygen saturation, heart rate, and BIS values did not differ significantly between the TCI and TIVA groups (*P=*0.090, *P=*0.416, and *P=*0.716, respectively). However, the mean BIS value at each time point was 1.417 units greater in the TIVA group than in the TCI group (Table 2).

When comparing the time intervals, the duration between infusion initiation and the start of surgery (6 minutes in the TIVA group and 8 in the TCI group; *P=*0.009) and that between the end of surgery and the time of transfer from the operating room demonstrated significant differences (7 minutes in the TIVA group and 10 in the TCI group; *P=*0.016; *P=*0.048, *P=*0.009, and *P=*0.016, respectively). The mean propofol consumption per unit time was 23.67±6.57 mg in the TIVA group. The TCI group demonstrated a significantly lower value at 20.69±4.52 mg. Although the total propofol consumption was slightly greater in the TIVA group than in the TCI group, the difference was not statistically significant (Table 2). The TIVA group demonstrated shorter intervals for both the onset of surgery and postoperative recovery in comparison to the TCI group (Table 2).

At the beginning of the surgical procedure, additional airway intervention was required in 15 patients in the TCI group and 19 in the TIVA group (no statistically significant difference). Following the intervention, oxygen saturation levels rose above 90% in all patients. Airway placement requirements did not differ significantly between the TIVA and TCI groups throughout the procedure. However, at the 10^th^ minute, 71.4% of patients in the TIVA group and 55.6% in the TCI group required airway support (Table 3). No significant difference was found between the TIVA and TCI groups in terms of the time-dependent trends of oxygen saturation, heart rate, or BIS values (*P=*0.090, *P=*0.416, and *P=*0.716, respectively). Although the mean BIS value at each time point was 1.417 units greater in the TIVA group than in the TCI group, this difference was not significant (Table 4).

Throughout the follow-up period, none of the patients experienced postoperative vomiting, delirium, or any major complications (Table 4).

## Discussion

This study revealed that in patients over 65 years of age, the time to surgical readiness and the post-procedure recovery times were significantly shorter in the TIVA group versus the TCI group. While the propofol consumption per unit time was lower in the TCI group, the total propofol consumption was similar between the groups. Although the need for airway placement (improvement in desaturation after airway placement) was greater in patients in the TIVA group, this difference was not significant.

Furthermore, patients receiving TIVA demonstrated a faster recovery following the discontinuation of propofol compared to those receiving TCI. Similarly, Mazanikov et al.^6^ reported longer recovery times in patients aged between 18 and 65 years using who underwent endoscopic retrograde cholangiopancreatography via TCI than in those who underwent patient-controlled sedation. In their study, recovery was 10±13 minutes with TCI and 5±6 minutes with patient-controlled methods.^6^ Similarly, Lehmann et al.^7^ observed shorter extubation times with manual infusion (11.9±2.4 minutes) versus TCI (15.6±6.8 minutes) in patients undergoing defibrillator implantation with low cardiac output. Conversely, in another study evaluating ERCP performed with laryngeal mask airway, TCI was associated with significantly faster recovery than TIVA (11.60±2.27 minutes vs. 15.4±3.25 minutes; *P *< 0.001).^8^ Passot et al.^9^ also demonstrated that although both groups had similar propofol consumption, TCI allowed for quicker recovery.

When we evaluated our results in terms of propofol consumption, the volume of propofol administered per unit of time was greater in the TIVA group (23.5 mg, *P=*0.048). Although the total dose of propofol administered did not differ significantly between the groups, it was numerically higher in the TIVA group (219.14±58.97 mg) compared to the TCI group (205.45±42.22 mg). The existing literature presents variable results on this topic. For instance, Mu et al.^10^ reported that pediatric patients in the TCI group received a larger propofol dose without any improvement in recovery time. In other studies conducted on adult patients, although propofol consumption was found to be slightly greater in the TCI group, the difference was not significant.^8, 9^ However, additional studies have demonstrated that propofol consumption decreases with increasing age.^3, 4^ Although the TCI method has been associated with increased propofol consumption in some studies, it has generally been linked to better-controlled sedation and shorter recovery times. Our findings showing lower total and per-minute propofol consumption in the TCI group are in line with prior observational studies focusing on elderly patients undergoing procedural sedation. For instance, a prospective study evaluating TCI sedation during gastrointestinal endoscopy in geriatric patients reported adequate sedation with favorable recovery and safety profiles, suggesting that TCI may offer a more efficient drug delivery tailored to patient needs, thereby avoiding over-sedation and minimizing propofol usage.^4^ Similarly, in a recent study evaluating propofol administration via TCI during gastrointestinal endoscopic procedures, Sarraj et al.^11^ reported that the propofol consumption per unit time was significantly lower in the TCI group compared to the nurse-administered intermittent bolus group (8.2±2.7 mg min^-1^ vs. 9.3±3.4 mg min^-1^; *P=*0.046). This observation is in line with the pharmacokinetic nature of TCI, where the infusion algorithm maintains a stable target effect-site concentration throughout the procedure, often resulting in slightly elevated per-minute infusion rates without increasing the total drug dose. This finding suggests that TCI systems may achieve the desired depth of sedation with more precise dosing and reduced drug requirements. The same study also reported a trend toward lower total propofol usage in the TCI group, which is consistent with the results observed in our study.

Apart from the higher prevalence of hypertension in the TIVA group (22 patients) compared to the TCI group (13 patients), the demographic and clinical profiles of patients were similar between groups. Moreover, at all recorded time points, the mean SBP was higher in the TIVA group compared to the TCI group. This observation may be attributed to the higher prevalence of hypertension among patients receiving TIVA. Moreover, fluctuations in blood pressure and transient hypertensive episodes occurred more frequently among patients receiving TIVA. In our study, both groups demonstrated a reduction in SBP compared with baseline values during the procedure. However, when analyzing the time-dependent trends, SBP values in the TCI group showed less fluctuation and remained closer to baseline levels compared to the TIVA group. This pattern supports the statement that SBP was “more stable” in the TCI group. The improved stability in the TCI group is likely attributable to the pharmacokinetic delivery algorithm of TCI, which maintains a consistent target effect-site concentration and avoids sudden peaks in plasma propofol levels. This contrasts with manually controlled infusion in TIVA, where bolus dosing may cause transient hemodynamic changes. While some studies have shown no clear hemodynamic advantage with TCI despite faster induction and recovery times,^8^ others support our findings. Similar to our findings, Wang et al.^12^ conducted a prospective randomized crossover trial in anaesthesiology residents performing colonoscopy sedation and reported that TCI of propofol provided greater hemodynamic stability, higher endoscopist satisfaction, and a shorter recovery time compared with manually controlled infusion, without increasing adverse events. These results support our observation that the modest advantages of TCI over conventional infusion may be particularly relevant in short-duration endoscopic procedures, especially when performed by less experienced anaesthesia providers.

Oxygen supplementation was provided to all patients through a facemask at a rate of 5 liters per minute. At the onset of surgery, airway adjuncts were required in 15 patients from the TCI group and 19 from the TIVA group—though this difference was not statistical significance. Following the intervention, oxygen saturation levels rose above 90% in all patients. Comparison of airway placement requirements at all time intervals revealed no significant differences between the TIVA and TCI groups. However, at the 10^th^ minute, airway placement was performed in 71.4% of patients in the conventional TIVA group and 55.6% in the TCI group. Although this difference was not significant, we considered that airway patency was better maintained in the TCI group. The fact that the rate of airway placement at the 10^th^ minute was higher than at other time points in both groups may have resulted from a decrease in the need for propofol due to a decrease in stimuli such as cystoscopy placement and positioning. Interestingly, another study reported lower SpO₂ values in patients sedated with TCI compared to TIVA.^12^ During anaesthesia induction, the administration of intravenous agents as a bolus leads to a more rapid achievement of peak plasma drug concentrations and faster attainment of threshold effect site concentrations. However, rapid anaesthesia induction may increase the risk of complications such as hemodynamic instability and apnea. Although no significant hemodynamic differences were observed in our study, the need for airway intervention was greater in the conventional TIVA group. This finding may be attributed to the rapid rise in the effect-site concentration of propofol in the conventional TIVA group, likely resulting from the use of bolus dosing.

The interval between anaesthesia induction and surgical initiation was found to be significantly shorter in the TIVA group. A similar observation was made by Hunt-Smith et al.,^13^ who compared TCI and manual infusion in 123 surgical patients and reported prolonged induction with TCI. Although the difference in total propofol consumption was not statistically significant, it tended to be lower in the TCI group. Furthermore, no significant variation in recovery times was noted between the two groups in that study.^13^ The rapid rise in the effect-site concentration of propofol observed in the conventional TIVA group was likely attributable to the use of bolus dosing.

In our study, the number of airway interventions was lower in the TCI group; however, this difference was not found to be statistically significant. Consistent with our observations, the literature also suggests that airway patency is maintained more effectively in the TCI group.  In a study conducted by Struys et al.,^14^ which included 90 female patients and compared the use of propofol administered via TCI and manual infusion, the number of patients who experienced apnea lasting longer than 20 seconds was significantly lower in the TCI group than in the manual infusion group. Additionally, Clouzeau et al.^15^ demonstrated that during fiberoptic bronchoscopy performed on patients with non-invasive ventilation, TCI not only preserved spontaneous breathing but also induced minimal alterations in hemodynamic status. While these observations suggest a potential advantage of TCI in maintaining airway patency, this finding in our study should be interpreted with caution and confirmed by larger-scale investigations.

In our study, BIS monitoring was used in both groups, and the duration spent below the lower sedation threshold value of 60 was minimal in both groups, with no significant difference. The lowest BIS recorded was 40. Liu et al.^16^ demonstrated that closed-loop infusion systems provide better control of BIS values than open-loop control systems do. In a study involving 200 patients undergoing upper gastrointestinal endoscopy, participants were divided into two groups based on whether BIS monitoring was utilized. The mean propofol infusion rate was significantly greater in the group without BIS monitoring. BIS monitoring not only reduced propofol consumption but also allowed the procedure to be performed safely.^3^ These findings highlight the importance of BIS monitoring as a valuable adjunct to optimizing propofol administration, enhancing patient safety, and potentially improving pharmacoeconomic effectiveness during sedation, particularly in elderly patients.

No major postoperative complications were observed in any of the patients during the initial 24-hour postoperative period. When evaluating minor complications, postoperative nausea was documented in three patients from the TIVA group and in one patient from the TCI group; this difference was not statistically significant. Moreover, none of the patients exhibited vomiting or postoperative delirium during the observation period.

### Study Limitations

Several limitations should be acknowledged. First, although the sample size was adequate for the primary outcomes, a larger cohort could enhance the statistical power and allow for more robust subgroup analyses. Second, the single-center design of the study may restrict the applicability of the results to broader clinical contexts or other healthcare institutions. Third, the procedural duration was narrowly defined between 7 and 12 minutes, which precludes assessment of sedation techniques in longer or more complex procedures. Lastly, while BIS monitoring was employed to ensure adequate sedation depth, additional parameters such as cognitive recovery scales or patient satisfaction scores were not evaluated.

## Conclusion

In this study comparing TCI and conventional TIVA for sedation in geriatric patients undergoing cystoscopy, both techniques were found to be safe and effective. Although the time from anaesthesia induction to surgical initiation and the recovery time were shorter in the TIVA group, the TCI group exhibited more stable hemodynamic parameters and lower propofol consumption. While airway interventions were less frequent in the TCI group, this finding needs to be supported by larger-scale, multicenter studies. BIS monitoring enabled adequate sedation depth in both groups; however, no significant reduction in propofol consumption was observed. No major postoperative complications, delirium, or significant differences in nausea and vomiting were observed between the groups. Given these findings, both sedation techniques appear to be clinically viable in geriatric patients; however, the choice of method may be guided by patient-specific factors such as cardiovascular stability and airway sensitivity. Further research involving longer surgical durations and larger, diverse patient populations is needed to validate and expand upon these results.

## Ethics

**Ethics Committee Approval:** The Human Research Ethics Committee of Ankara University Faculty of Medicine approved this study (approval no.: İ10-611-22, date: 10.11.2022).

**Informed Consent:** Patients were informed about participation in the study before the procedure, and written informed consent was obtained from all participants.

## Figures and Tables

**Table 1. Baseline Demographic and Clinical Characteristics of Patients in the TIVA and TCI Groups:** 

Variables		TIVA (n = 30)	TCI (n = 30)	Total (n = 60)	*P *value
Age	Median (min-max)	70.50 (65.00-87.00)	69.00 (65.00-81.00)	69.00 (65.00-87.00)	0.229^b^
BMI	Mean ± SD	26.67±3.44	26.30±3.08	26.49±3.25	0.663^a^
ASA score, n (%)	I	1 (3.3)	2 (6.7)	3 (5.0)	0.677^d^
	II	18 (60.0)	20 (66.7)	38 (63.3)	
	III	11 (36.7)	8 (26.6)	19 (31.7)	
DM, n (%)		14 (46.7)	10 (33.3)	24 (40.0)	0.292^c^
Hypertension, n (%)		22 (73.3)	13 (43.3)	35 (58.3)	0.018^c^
ASHD, n (%)		12 (40.0)	11 (36.7)	23 (38.3)	0.791^c^
Asthma, COPD n (%)		2 (6.7)	2 (6.7)	4 (6.7)	1.000^d^
CVD, n (%)		2 (6.7)	1 (3.3)	3 (5.0)	1.000^d^
Cancer, n (%)		13 (43.3)	10 (33.3)	23 (38.3)	0.426^c^

^a^Student’s t-test; ^b^Mann-Whitney U test; ^c^Chi-square test; ^d^Fisher’s exact test

TCI, target-controlled infusion; TIVA, total intravenous anaesthesia; SD, standard deviation; min, minimum; max, maximum; BMI, body mass index; ASA, American Society of Anesthesiologists; DM, diabetes mellitus; ASHD, arteriosclerotic heart disease; COPD, chronic obstructive pulmonary disease; CVD, cerebrovascular disease

**Table 2. Procedural Parametres During Sedation TIVA and TCI Groups:** 

Variables		TIVA (n = 30)	TCI (n = 30)	Total (n = 30)	*P *value
Total surgery time (minute)	Median (min-max)	8.50 (6.00-12.00)	10.00 (7.00-13.00)	10.00 (6.00-13.00)	0.131^b^
Time between infusion start/surgery start (minute)	Median (min-max)	6.00 (3.00-15.00)	8.00 (5.00-14.00)	7.00 (3.00-15.00)	0.009^b^
Time between end of surgery/exit from OR (minute)	Median (min-max)	7.00 (3.00-13.00)	10.00 (4.00-14.00)	8.00 (3.00-14.00)	0.016^b^
Propofol consumption (mg)	Mean ± SD	219.14±58.97	205.45±42.22	212.30±51.32	0.511^a^
Propofol consumption per unit of time (mg)	Median (min-max)	23.50 (14.20-46.25)	21.33 (14.04-31.71)	22.33 (14.04-46.25)	0.048^b^
Time to stay under BIS 60 (minute)	Median (min-max)	2.00 (0.00-8.00)	0.00 (0.00-13.00)	2.00 (0.00-13.00)	0.710^b^
BIS value	Mean ± SD	66.19±7.48	65.38±6.53	65.79±6.97	0.656^a^

^a^Student’s t-test; ^b^Mann-Whitney U test

TCI, target-controlled infusion; TIVA, total intravenous anaesthesia; SD, standard deviation; min, minimum; max, maximum; OR, operating room; mg, miligram

**Table 3. Frequency of Airway Interventions at Different Procedural Time Points in TIVA and TCI Groups:** 

Times	TIVA		TCI		*P *value
	n	%	n	%	
Surgery start	19	63.3	15	50.0	0.297^a^
2 min	20	66.7	15	50.0	0.190^a^
4 min	21	70.0	15	50.0	0.114^a^
6 min	21	70.0	15	50.0	0.114^a^
8 min	21	72.4	14	48.3	0.060^a^
10 min	10	71.4	10	55.6	0.358^a^
Surgical end	22	73.3	15	50.0	0.063^a^

^a^, chi-square test; ^b^, Fisher’s exact test; TCI, target-controlled infusion; TIVA, total intravenous anaesthesia; min, minimum

**Table 4. Postoperative Outcomes in TIVA and TCI Groups:** 

Variables	TIVA (n = 30)	TCI (n = 30)	Total (n = 30)	*P *value
Nausea, n (%)	3 (10.0)	1 (3.3)	4 (6.7)	0.612^b^
Vomiting, n (%)	0	0	0	-
Delirium, n (%)	0	0	0	-
Major postoperative complication (%)	0	0	0	-

^b^Fisher’s exact test; TCI, target-controlled infusion; TIVA, total intravenous anaesthesia
